# Experimental Study on the Effect of Micro-Texture on EHL Point-Contact Film Thickness Subject to Sliding Conditions

**DOI:** 10.3390/ma15227926

**Published:** 2022-11-09

**Authors:** Jianxin Sun, Linqing Bai, Feng Guo, Zulfiqar Ahmad Khan

**Affiliations:** 1 School of Mechanical and Automotive Engineering, Qingdao University of Technology, Qingdao 266520, China; 2 Department of Design and Engineering, NanoCorr, Energy & Modelling (NCEM) Research Group, Bournemouth University, Dorset BH12 5BB, UK

**Keywords:** laser surface texturing, EHL, point contact, film thickness, sliding conditions

## Abstract

Processing texture on contact surfaces can improve the friction performance of mechanical comments. In this research, micro-dimple textures with various parameter were processed on a steel ball’s surface with a picosecond laser. Then, the EHL (elastohydrodynamic lubrication) oil film thickness was measured on an optical ball-on-disc tribometer subjected to pure sliding conditions. The effects of sliding velocity, load, dimple location and dimple depth on the film thickness were analyzed. The results showed that the dimple affected the pressure distribution in the contact area, which in turn changed the distribution of the local film thickness. An increase in the local film thickness occurred between the dimple and outlet of the contact region, while a decrease in the film thickness formed from the dimple to the entrance of the contact area and both sides of the dimple’s edge. Velocity and applied loads affected the film thickness distribution as well. As the sliding velocity increased, the film thickness increasing region enlarged, while the film thickness-decreasing area shrank. The increase in load resulted in a negative effect on the increase in film thickness. This study will provide a reference for point-contact designs with low friction conditions.

## 1. Introduction

Improving the friction performance of contact surfaces is important to enhance mechanical efficiencies. As an effective way to reduce friction and wear, surface texturing has received extensive attention. It has been widely used in mechanical seals [1,2,3,4], engine pistons [5,6], antifriction bearings [7,8,9] and other practical engineering fields. There are various texturing mechanisms with different lubrication states. It is generally believed that texturing can store wear debris and reduce surface scratches under dry friction conditions. The lubricant is stored in the texture and then is squeezed out to replenish the contact area when the contact pairs are deformed under the state of boundary and mixed lubrication. Under hydrodynamic lubrication, hydrodynamic pressure effects formed to enhance the bearing capacity and reduce wear.

Research on texture mainly focused on face-to-face contact in the early stage and obtained excellent friction and wear reduction effects compared with untextured surfaces [1,6,10]. The role of surface textures and the corresponding lubrication mechanisms of face-to-face friction pairs with low contact pressures has received some exploration. However, studies on the friction performance of texture under point-contact conditions number relatively few. Intuitively, the existence of textures under point contact will increase the contact pressure and affect the fatigue life of mechanical components. However, via experiments and numerical calculations, researchers found that appropriate texture morphology also had a beneficial impact on the contact pairs under sliding/rolling motion [11,12]. Hirayama et al. [13] machined texture on steel balls and measured the EHL oil film thickness. They found that texture with nano-depths could increase the film thickness relative to elastohydrodynamic lubrication under pure rolling conditions. The experimental results of Boidi et al. [14] showed that under mixed lubrication, textures could reduce the friction force by 20%, but it had little effect on lubrication performances under full-film lubrication. Křupka et al. [15] investigated the effect of micro-textures on the lubricating performance of steel ball surfaces at instantaneous speeds. The results showed that the lubricant stored in the micro-dents on the ball’s surface contributed to the oil film formation and improved the lubricating efficiency. Mourier et al. [16,17,18] found that the local film thickness reduced if the texture depth in the contact region was larger. With the decrease in texture depth, the decreasing film thickness became weaker, and the texture gradually became beneficial for the formation of film thickness. Hsu et al. [19] conducted a bearing life test, and the results showed that the combination of machining pit textures on the bearing’s raceway and using ZDDP additives increased the bearing’s fatigue life by three times.

Most research work on the influence of texture on point contact under the EHL state was outperformed at a relatively small slip–roll ratio, but there are a few studies on pure sliding conditions. Moreover, the texture position in the contact area has different effects on lubrication performance, but there are not many studies in the literature investigating this phenomenon. This work aims at researching the influence of dimple textures on the formation of lubricating oil films under pure sliding conditions. The influence of texture parameters, texture positions, sliding speeds and load on point-contact EHL film thickness will be studied and discussed. This research will contribute to the potential applications of textures under EHL lubrication conditions in practical projects.

## 2. Experimental Details

### 2.1. Test Rig

**Figure 1:**
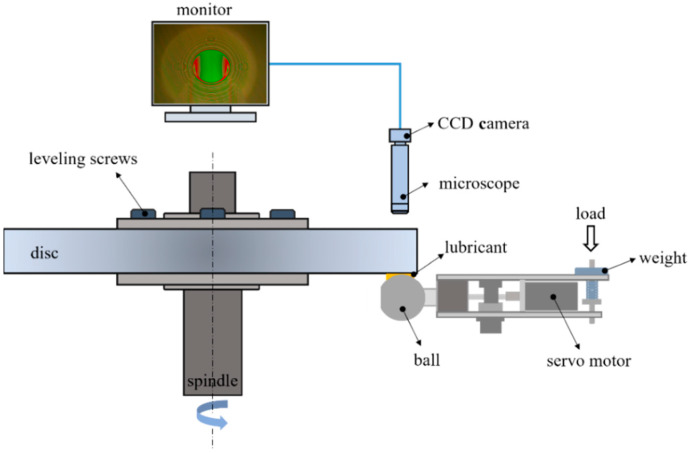
Schematic of the test rig.

A ball-on-disc test apparatus independently built in a laboratory was used to carry out all experiments under various operating conditions. Figure 1 presents the test apparatus’ schematic. A glass disc and steel ball are fixed to their respective shafts, which can be driven by a motor to research the set speed. The load is applied to the ball drive by a weight. Meanwhile, a CDD camera is used to collect the interference images, and DIIM [20] software independently developed in the laboratory can process the obtained optical interference images to obtain the corresponding thickness of lubricated oil films. The glass disc is supported by six leveling screws, so the glass disc can be adjusted to a relatively flat operating state. This is very important to ensure the smooth operation of the glass disc and the corresponding stability of the test’s results. 

### 2.2. Sample

**Figure 2:**
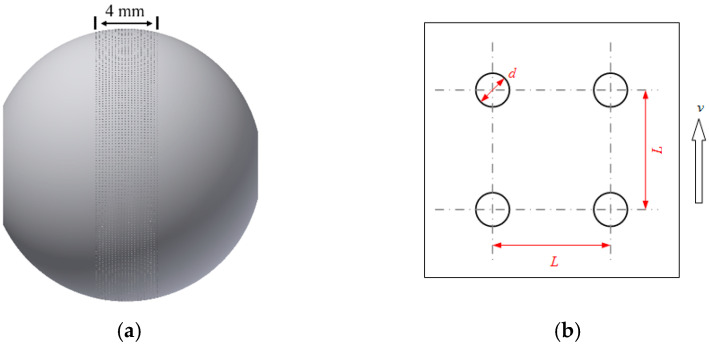
Circle-dimple textured ball: (**a**) 3D schematic diagram and (**b**) texture parameter diagram.

To explore the lubrication performance of textured surfaces under EHL lubrication conditions, the film thickness of untextured balls and circle-dimple textured balls with various geometrical parameters were measured and compared. As presented in Figure 2, a circle-dimple region with width of 4 mm distributes uniformly along the circumferential direction of the ball with a dimple diameter of *d*, depth of *h_d_*, center distance of *L* and area ratio of *S_d_*.

**Figure 3:**
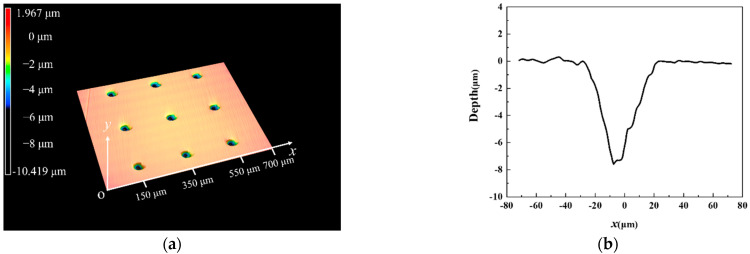
Topography of textured steel ball surface: (**a**) 3D topography and (**b**) 2D profile.

Table 1 presents the sample parameters used in this research. The outside diameter of the K9 glass disc is 150 mm with a roughness of *Ra* ≈ 20 nm. To obtain clear interference images, a 20 nm-thick chromium layer was coated on the working surface of the glass disk. For the steel ball, the diameter is 25.4 mm and the roughness is *Ra* ≈ 20 nm. Before tests, textures were fabricated on the ball’s surface along the circumference of the rotation by a picosecond laser. The laser’s processing parameters are presented in Table 2. Textures with different depths can be obtained by controlling the laser’s processing times. After laser processing, burrs around the texture were carefully removed by polishing. Table 3 presents textures parameters that have been used in this research study. Sample 3 represents the untextured steel ball. The morphology of laser texturing surface was measured by using a confocal laser scanning microscope (VK-X1000, Keyence, Osaka, Japan) as shown in Figure 3.

### 2.3. Experimental Protocol

Before testing, both the glass disc and balls were cleaned with petroleum ether and ethyl alcohol, respectively. Then, 1 mL synthetic base oil PAO 20 (provided by Shanghai NacoLubrication Corporation, Shanghai, China) with viscosities of 385.7 mPa·s at 21 °C (tested by rheometer MCR302, Anton Paar, Graz, Austria) was injected between the glass disc and the ball as a lubricant for each test. By controlling the servo motor, the glass disc rotated from 32 mm/s to 512 mm/s with the setting speed interval and the running time lasted for 10 s under each rotational speed. The steel ball remained stationary to achieve pure sliding motions. Three loads of 8.3 N, 16.7 N and 25 N were applied by weights. During the tests, an optical interference image was collected via a CCD camera. When one test was completed, both the glass disc and ball were cleaned again. Then, the ball rotated with an angle to a new position for the next set of tests. This is very important for ensuring that all repeated tests were carried out with the same texture parameters. The experimental conditions are presented in Table 4.

## 3. Experimental Results

### 3.1. Effect of the Sliding Speed

**Figure 4:**
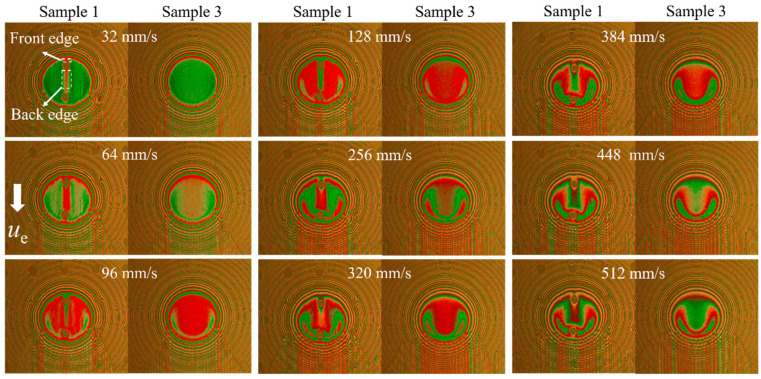
Interference images of sample 1 and sample 3 at different sliding speeds (load = 16.7 N).

Figure 4 shows a comparison of the interference images of sample 1 and sample 3 (untextured steel ball) at different sliding speeds when the dimple is located at the entrance of the hertz contact region under a load of 16.7 N. The white arrow presented the flow direction from the entrance to the outlet in the contact region. For a clear description, the dimple edge close to the entrance of the hertz contact area was defined as the front edge, and the other side, which was close to the outlet, was defined as the back edge. As shown in Figure 4, at the back edge of the dimple, a local increasing film thickness region formed, and at both sides of the dimple, a reducing film thickness region formed.

**Figure 5:**
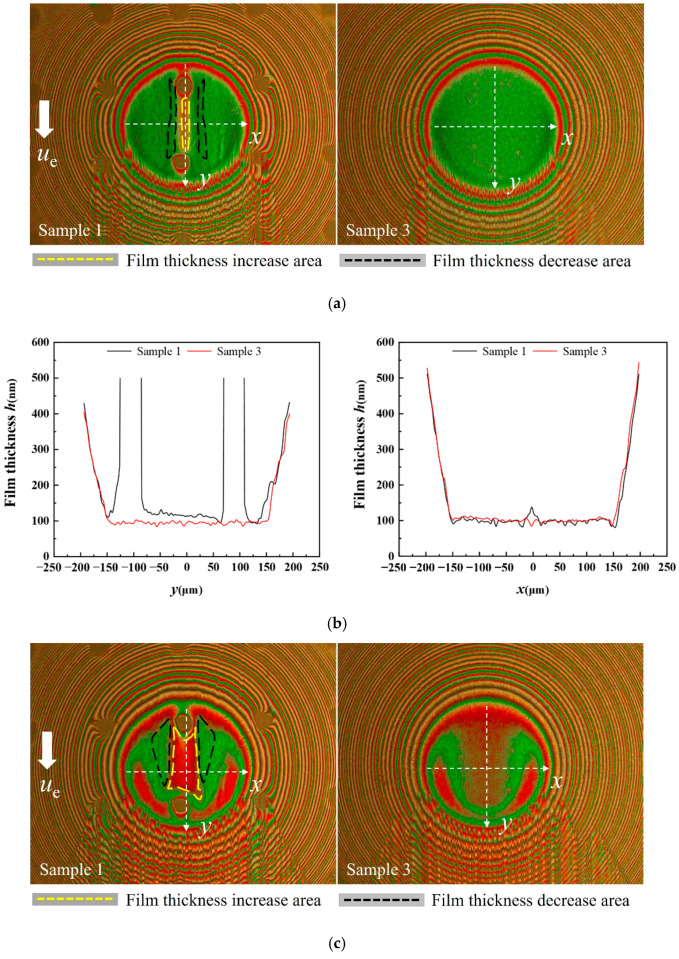

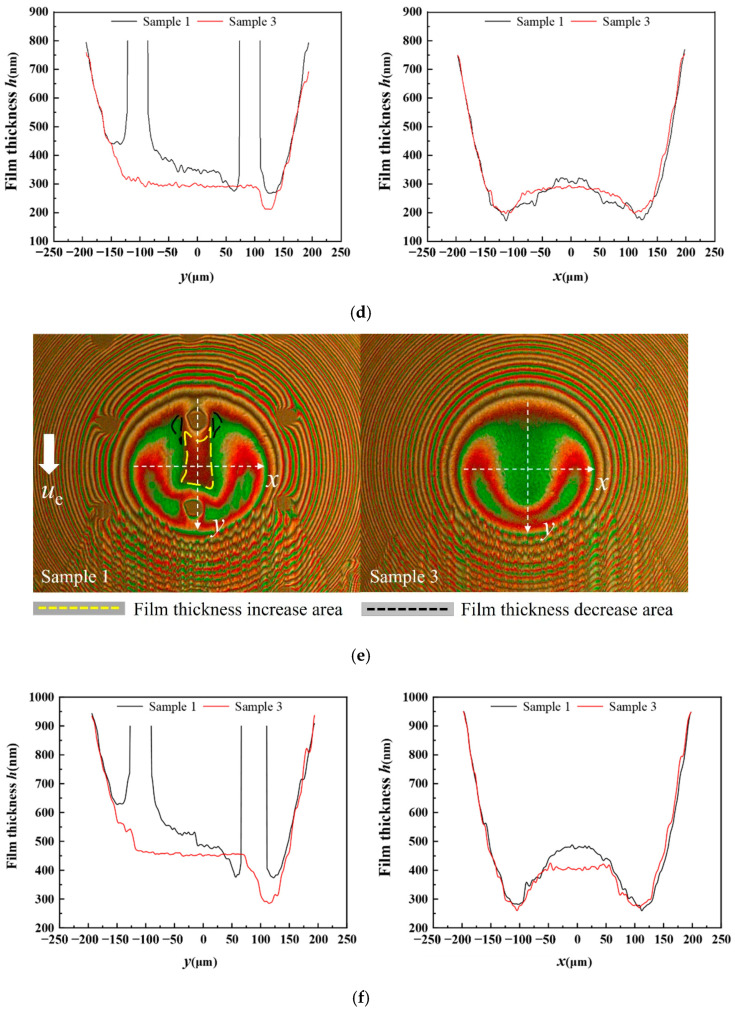
Film thickness distribution and profile of sample 1 and sample 3 along the *x* and *y* axes (load = 16.7 N): (**a**) distribution of film thickness—32 mm/s; (**b**) film thickness profile along *x* and *y* directions—32 mm/s; (**c**) distribution of film thickness—256 mm/s; (**d**) film thickness profile along *x* and *y* directions—256 mm/s; (**e**) distribution of film thickness—512 mm/s; and (**f**) film thickness profile along *x* and *y* directions—512 mm/s.

The film thickness profiles of sample 1 and sample 3 along the *x* and *y* directions at three different speeds were presented to further analyze the influence of sliding speeds on film thickness distributions. The results are shown in Figure 5, where *x* and *y* axes were perpendicular and parallel to the sliding velocity, respectively. It should be noted that since the depth of the dimples was much larger than the film thickness, the morphology of the texture could not be fully shown in the contour figure. Two vertical lines were used to represent the texture edge in Figure 5. Moreover, the center distance between two dimples was 200 μm while the diameter of hertz contact area was close to 200 μm under a load of 16.7 N, which led to the existence of two dimples in the contact area simultaneously with one of the dimples located at the entrance of the contact region. The influence of the dimple located at the entrance of the contact region was discussed and the other was ignored. The change in film thickness could be judged by different interference fringe orders in the interference images. The area outlined by yellow and black dashed lines in Figure 5 represents the increasing film thickness areas and decreasing film thickness areas, respectively. When the sliding speed was 32 mm/s, a narrow increasing film thickness region formed at the back edge of the dimple and two sides. Comparing the profile of the film thickness along the *x* axis in Figure 5b, the decreasing film thickness region was not as obvious as the increasing film thickness region. When the sliding speed was 256 mm/s, the increasing film thickness area enlarged, and the decrease in film thickness at both sides of the dimple became more apparent. When the sliding speed was 512 mm/s, the proportion of the increasing film thickness area remained almost unchanged, but the decreasing film thickness area became smaller and only presented at both sides of the dimple, as shown in Figure 5e.

**Figure 6:**
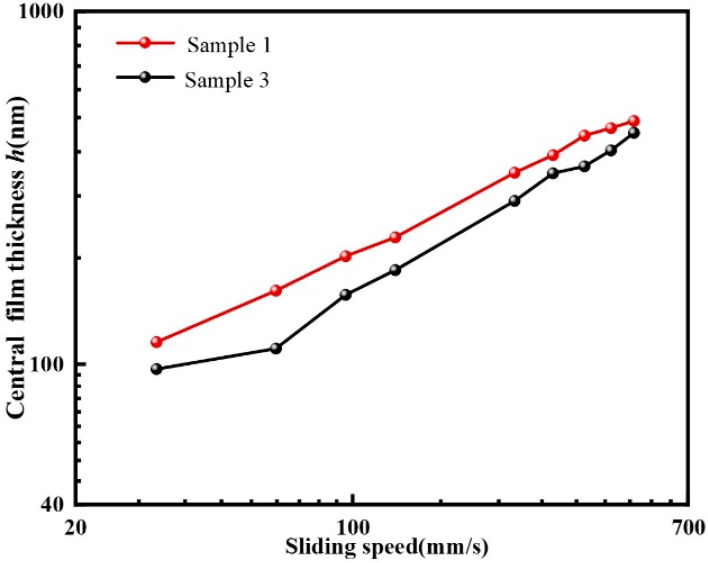
Comparison of central film thickness of sample 1 and sample 3 (load = 16.7 N).

To intuitively express the oil film thickness increase at the back edge of the texture, Figure 6 compares the central film thickness of sample 1 at the junction of the *x* and *y* axes with sample 3. In general, the increase in film thickness continuously enhanced with increasing sliding velocities. The central film thickness of textured ball was higher than the untextured steel ball under the same sliding speeds in the experiment.

### 3.2. Effect of the Load

**Figure 7:**
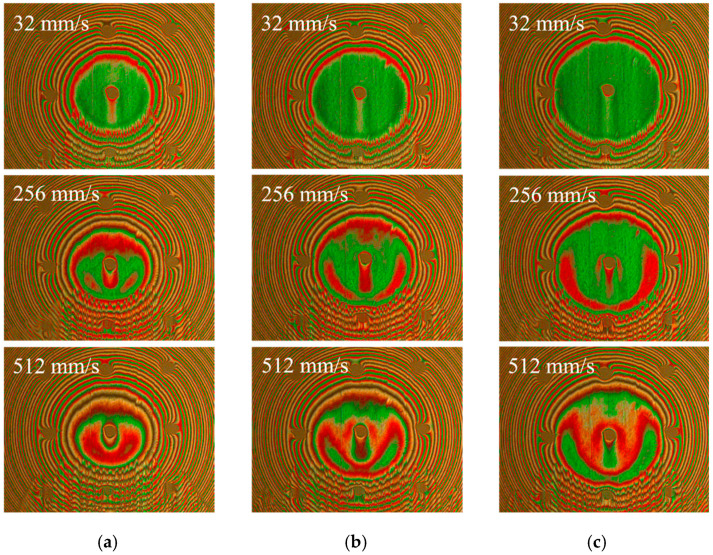
Interference images of sample 1 with dimple located at the middle of contact area under different loads: (**a**) 8.3 N; (**b**) 16.7 N; (**c**) 25 N.

**Figure 8:**
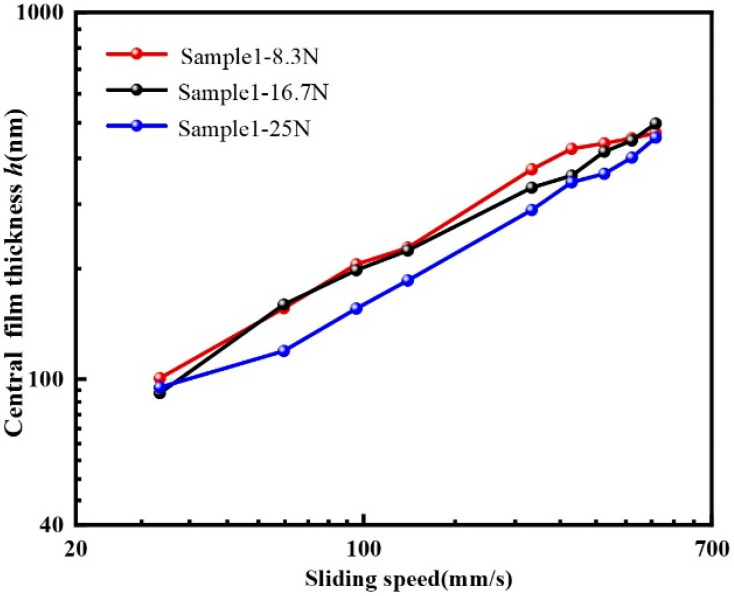
Comparison of central film thickness of sample 1 under different loads.

The interference images and film thickness of sample 1 under the loads of 8.3 N, 16.7 N and 25 N were collected and analyzed when the texture was in the middle of the contact region to explore the influence of loads on EHL film thickness. Figure 7 and Figure 8 show the interference images and central film thickness comparison for sample 1 under different loads at three sliding speeds. In general, the film thickness increase at the back edge of the dimple lower, and the decrease in film thickness at both sides of the dimple tended to be more significant with increasing loads. However, the central oil film thickness was still higher than that of the untextured steel ball at the back edge of the dimple.

### 3.3. Effect of the Texture Location

**Figure 9:**
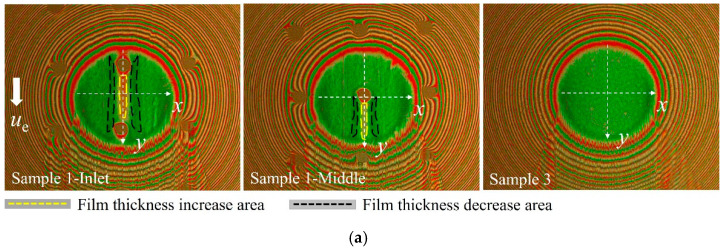

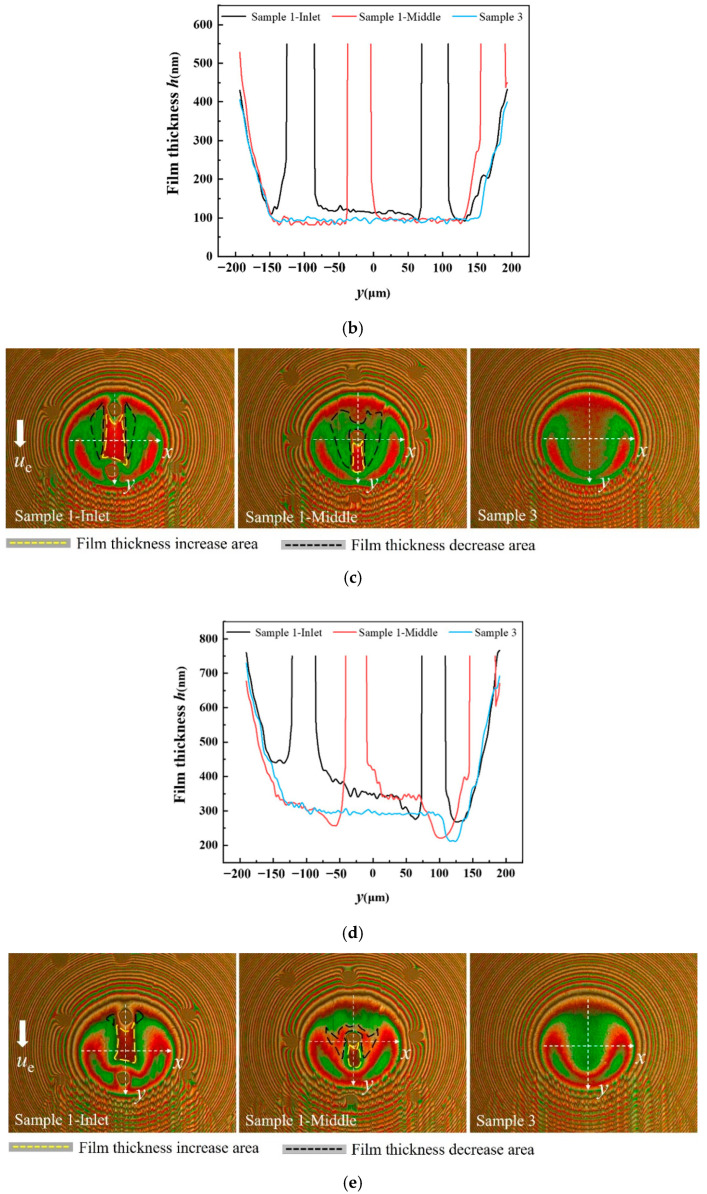

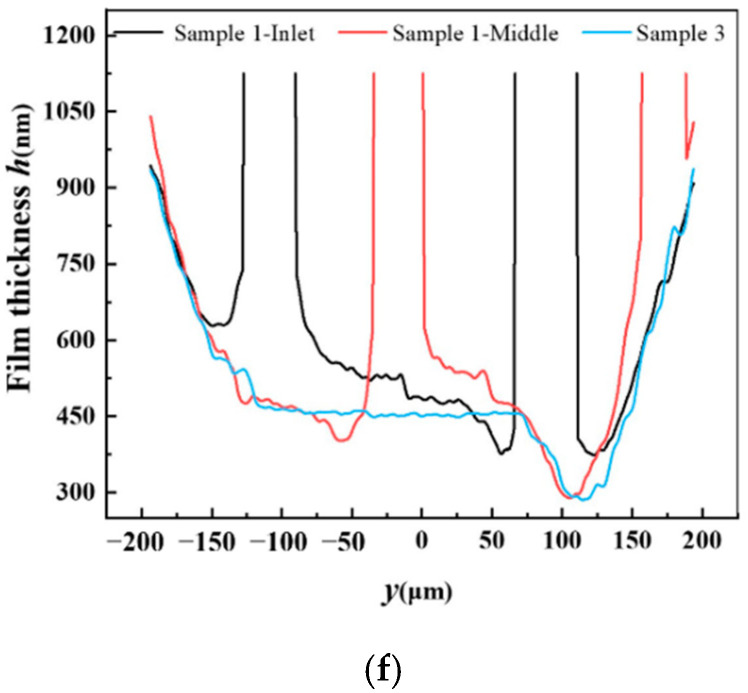
Film thickness profiles of sample 1 and the untextured ball along the sliding direction (load = 16.7 N): (**a**) distribution of film thickness—32 mm/s; (**b**) film thickness profile along the *y* direction—256 mm/s; (**c**) distribution of film thickness—256 mm/s; (**d**) film thickness profile along the *y* direction—256 mm/s; (**e**) distribution of film thickness—512 mm/s; and (**f**) film thickness profile along the *y* direction—512 mm/s.

The effect of the dimple’s position on film thickness was explored by changing the dimple’s location. The position of the dimple in sample 1 was adjusted relative to the entrance and the center of the contact area. Figure 9 compares the interference images and film thickness profiles of sample 1 along the sliding direction with the untextured ball under a load of 16.7 N.

Compared with dimples located at the entrance of the contact region, the increasing local film thickness area still formed at the back edge of the dimple and the decreasing film thickness area also formed on both sides of the dimple when the dimple was located at the middle of contact region. The difference was that there was a decreasing film thickness area at the dimple’s front edge. When the sliding speed was 32 mm/s, the film thickness profile along the sliding direction showed that the film thickness increased at the back edge and was not obvious compared with the dimple located at the middle of the contact region. As the sliding speed changed to 256 mm/s, a larger region of the film’s thickness-reducing area formed at the front edge and both sides of the dimple. It can be seen clearly from the interference image in Figure 9c. The increasing film thickness area was relatively small. A reduction in film thickness at the dimple’s front edge can be seen clearly from the film thickness profile in Figure 9d. When the sliding speed was 512 mm/s, the reducing film thickness area decreased, which was similar to that as the texture located at the entrance of the contact region. However, the decrease in film thickness at the front edge of the dimple still exists, and the decrease on both sides is larger.

### 3.4. Effect of the Texture Depth

**Figure 10:**
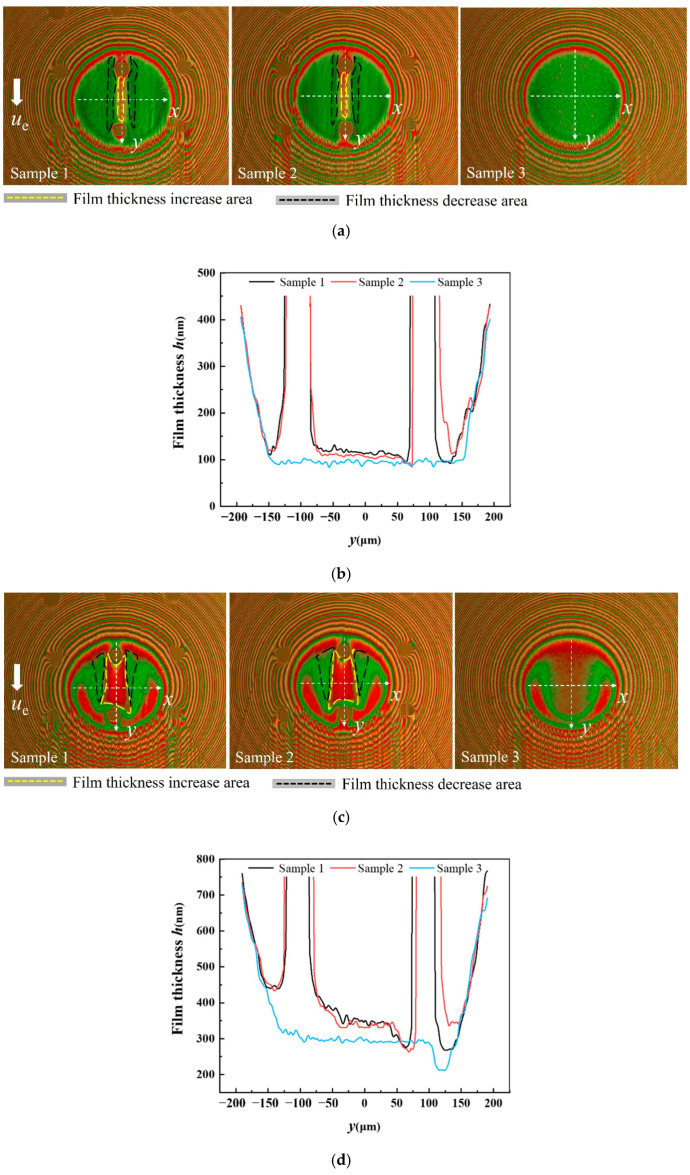

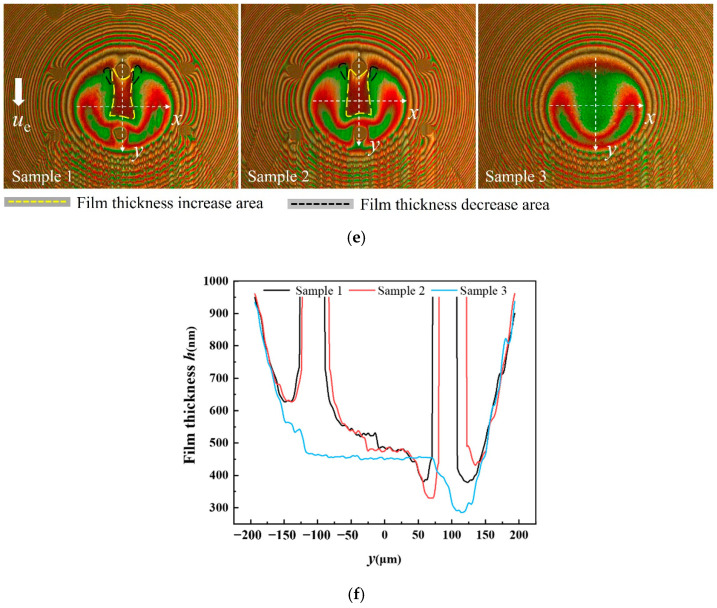
Film thickness profile of sample 1 and sample 2 with textures located at the entrance of contact region: (**a**) distribution of film thickness—32 mm/s; (**b**) film thickness profile along direction—32 mm/s; (**c**) distribution of film thickness—256 mm/s; (**d**) film thickness profile along the *y* direction—256 mm/s; (**e**) distribution of film thickness—512 mm/s; and (**f**) film thickness profile along the *y* direction—512 mm/s.

**Figure 11:**
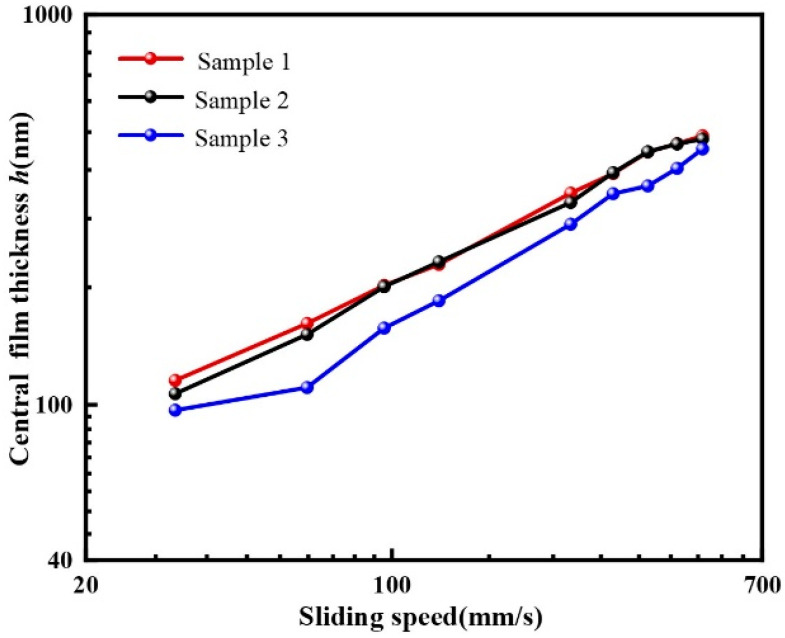
Central film thickness of sample 1 and sample 2 with textures located in the contact area.

Depth is an important factor affecting the frictional performance of texture [21]. Different texture depths will affect the pressure level inside the texture. To explore the dimple’s depth effect on oil film thickness, samples with different depths of 3 μm (sample 1) and 7 μm (sample 2) were used to carry out experiments. Both dimples had the same diameters and were located at the entrance of the contact region. The interference images and film thickness profiles under the load of 16.7 N are presented in Figure 10. As the interference images show, there was no obvious difference between sample 1 and sample 2. The film thickness profile of sample 1 was slightly higher than that of sample 2. Figure 11 shows the comparison of the central film thickness of sample 1, sample 2 and sample 3. The captured pictures of three samples in Figure 10 were at the same position in the contact region. The film thickness of sample 1 and sample 2 was very close, and the central film thickness of the two textured balls was higher than the untextured one.

## 4. Discussions

According to this research study, dimples in the contact area have two different effects on the film thickness, and these manifested as an increase in film thickness at the back edge of the dimple and a decrease in film thickness at the front edge and both sides of the dimple.

**Figure 12:**
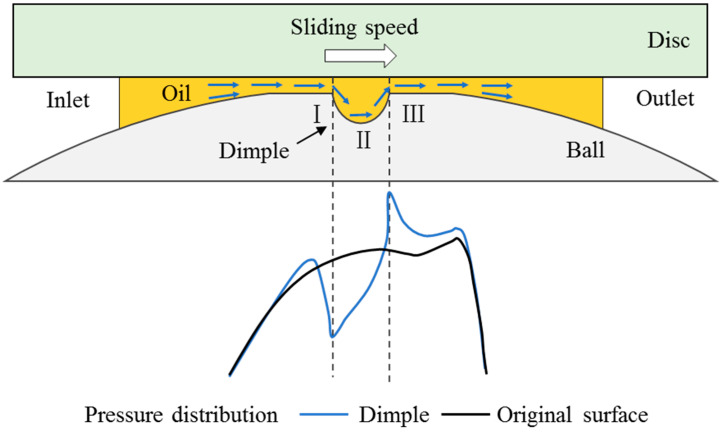
Schematic of the process of lubricating oils flowing through the dimple.

In fact, the existence of dimples in the contact area mainly affects local pressure distributions. The process of the flow of lubricant through a dimple can be separated into three stages, as shown in Figure 12. (1) Stage I: Driven by the glass disc, the lubricating oil flows into the contact area. When it flows to the front of the dimple, the pressure drops abruptly. In order to ensure the continuity of the flow, a pressure peak forms at the front of the abrupt pressure position, leading to a decrease in film thickness at the front edge of the dimple, which is similar to the formation of the secondary pressure peak of EHL. When the dimple is located at the entrance of the contact region, the pressure drop is not that significant as the dimple located at the middle of the contact region. When the dimple moves closer to the entrance, the pressure distribution becomes more similar to the normal hertz pressure distribution. Therefore, there is little or no decrease in film thickness at the front edge when the dimple is located at the entrance of the contact region. (2) Stage II: The surrounding pressure will be relatively high due to the existence of pressure drops in the dimple. As a result, the lubricating oil is driven to the dimple by the surrounding high pressure. This increases the pressure of the oil in the dimple, which produces an increase in oil viscosity. High-viscosity lubricating oils inside the dimple have difficulty flowing outward. (3) Stage III: The oil flows outside the dimple towards the outlet under the shearing action of the disk. A local elastic deformation area forms at the back edge of the dimple under the extrusion action of high-viscosity lubricating oils. In the end, an increasing film thickness area forms.

In the test, it can be seen that the sliding speed affects the film thickness’ distribution. The increase in lubricating oil viscosity is the main reason for the increase in film thickness at the back edge of the dimple, as previously stated. At a lower sliding speed, lubricating oils move from the tail of the dimple’s back edge towards the outlet, forming a narrow and convergent strip-shaped film-thickness-enhanced area. As the sliding speed increases, the amount of lubricant drawn out from the dimple also increases, causing and increasing local elastic deformations at the back edge of the dimple. This extends the increasing film thickness area along the sliding direction, and the average film thickness becomes higher. Moreover, when the lubricant flows out of the texture, the hydrodynamic effect formed at the convergence wedge, which promotes an increase in film thickness.

The depth of textures is a key factor affecting frictional properties as it affects the pressure and film thickness distribution in the contact region [21]. However, the film thickness did not show many differences as the dimple’s depth changed from 3 μm to 7 μm in this experiment. This may be due to the fact that the texture depth of 3 μm is too deep and a further increase in depth cannot produce obvious changes in pressure and corresponding film thickness. Limited by the laser processing equipment, only deeper textures can be processed on the ball for the time being. It is necessary to study the effect of shallower textures on film thickness in subsequent studies to deeply explore the influence of dimple depths on oil film thickness.

## 5. Conclusions

In this paper, the influence of dimple textures on EHL film thickness under pure sliding conditions was studied. The conclusions are listed below.

The dimple in the contact region affected the distribution of pressure and film thickness. Film thickness increased at the back edge of the dimple, but it decreased at both sides and the front edge of the dimple.

Sliding speeds and loads affected the film thickness distribution. Higher sliding speeds enlarged the increasing film thickness region and decreased the film thickness reduction area. The increase in load would cause a reduction in the increasing film thickness area and enhance the decrease in film thickness. 

Dimple locations had a significant influence on the film thickness distribution. The increasing film thickness area formed at the back edge of the dimple. There was either a smaller decreasing thickness area or no decreasing film thickness area when the dimple was close to the entrance of the contact area, and the decreasing film thickness area formed at the front and both sides of the dimple when the dimple was located at the middle of contact region.

The dimple’s depth did not show many differences on film thickness in this research study. This may be due to the lack of significant pressure variations at this depth range. Further exploration should utilize wider dimple depth ranges.

The presented results showed that processing dimples on the steel ball can enhance or reduce the local film thickness. Careful designs are needed to improve the performance of the texture under the point-contact conditions to meet the requirements of practical engineering applications.

## Figures and Tables

**Table 1:** Sample parameters.

	Material	Elastic-Modulus (GPa)	Poisson’s Ratio
Ball	52100 steel	210	0.3
Disc	K9 glass	81	0.29

**Table 2:** Laser processing parameter.

Wavelength	Pulse Energy	Pulse Frequency	Scanning Speed
532 nm	0.3 w	100 kHz	100 mm/s

**Table 3:** Parameters of samples.

Sample Number (No.)	Dimple Diameter *d*/μm	Dimple Depth *h*/μm	Area Ratio *S*_G_	Center Distance *L*/μm
1	45	3	0.16	200
2	45	7	0.16	200
3	/	/	/	/

**Table 4:** Parameters of samples.

Load (N)	Sliding Speed (mm/s)	Maximum Contact Pressure (MPa)	Slide/Roll Ratio	Temperature (°C)
8.3	32, 64, 96, 128, 256, 384, 512	337.2	2	21 ± 0.5
16.7		424.9		
25		486.3		

## Data Availability

The data are not publicly available due to ongoing work.
